# Nanozymes based on octahedral platinum nanocrystals with {111} surface facets: glucose oxidase mimicking activity in electrochemical sensors

**DOI:** 10.1007/s00604-023-05992-9

**Published:** 2023-09-30

**Authors:** Elisabetta Mazzotta, Tiziano Di Giulio, Valentina Mastronardi, Rosaria Brescia, Pier Paolo Pompa, Mauro Moglianetti, Cosimino Malitesta

**Affiliations:** 1 Laboratorio di Chimica Analitica, Dipartimento di Scienze e Tecnologie Biologiche e Ambientali (Di.S.Te.B.A.), Università del Salento, 73100 Lecce, Italy; 2 Istituto Italiano di Tecnologia, Nanobiointeractions & Nanodiagnostics, Via Morego 30, 16163 Genova, Italy; 3 Electron Microscopy Facility, Istituto Italiano di Tecnologia, Via Morego 30, 16163 Genova, Italy; 4 Istituto Italiano di Tecnologia, Centre for Cultural Heritage Technology (CCHT@Ca’ Foscari), Via Torino 155, 30172 Venice, Italy; 5 HiQ-Nano srl, Via Barsanti, 1, 73010, Arnesano (LE), Italy

**Keywords:** Octahedral platinum nanocrystals, Artificial enzymes, Pt nanozymes, Glucose-mimicking, Glucose sensor, Saliva hyperglycemia, Multiple pulse amperometry

## Abstract

The ability of shape-controlled octahedral Pt nanoparticles to act as nanozyme mimicking glucose oxidase enzyme is reported. Extended {111} particle surface facets coupled with a size comparable to natural enzymes and easy-to-remove citrate coating give high affinity for glucose, comparable to the enzyme as proven by the steady-state kinetics of glucose electrooxidation. The easy and thorough removal of the citrate coating, demonstrated by X-ray photoelectron spectroscopy analysis, allows a highly stable deposition of the nanozymes on the electrode. The glucose electrochemical detection (at −0.2 V vs SCE) shows a linear response between 0.36 and 17 mM with a limit of detection of 110 μM. A good reproducibility has been achieved, with an average relative standard deviation (RSD) value of 9.1% (*n* = 3). Similarly, a low intra-sensor variability has been observed, with a RSD of 6.6% (*n* = 3). Moreover, the sensor shows a long-term stability with reproducible performances for at least 2 months (RSD: 7.8%). Tests in saliva samples show the applicability of Pt nanozymes to commercial systems for non-invasive monitoring of hyperglycemia in saliva, with recoveries ranging from 92 to 98%.

**Graphical Abstract:**

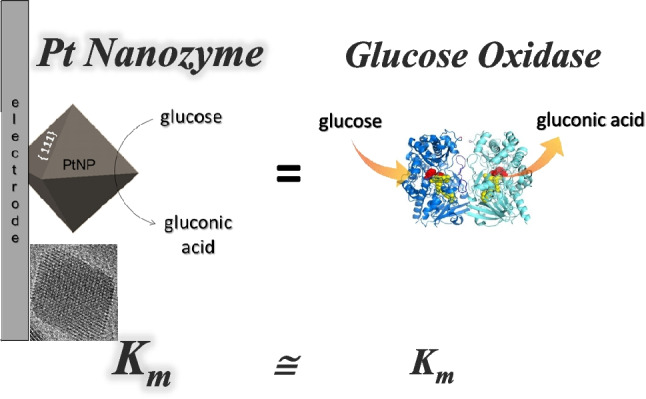

**Supplementary Information:**

The online version contains supplementary material available at 10.1007/s00604-023-05992-9.

## Introduction

Nanozymes are nanomaterials with intrinsic enzyme-like features [1–3]. Their ability to overcome the limitations of natural enzymes such as poor stability and high production cost has attracted great attention [4–6]. Due to the enormous progress in the synthetic methodologies [7], it is now possible to synthesize nanomaterials with tailor-made physico-chemical properties and catalytic activities allowing their application as nanozymes in a broad range of fields [8–10]. Acting as artificial enzymes, nanozymes offer several advantages, such as low cost, high stability, ease modification, and keeping high catalytic activity. These fascinating properties justify their large use in biosensing, environmental protection, and clinical fields [11, 12]. In this contest, nanoparticles composed of highly catalytic materials such as Pt [13–15] have received strong attention, especially in the electrocatalytic detection of small molecules such as glucose [16] due to their well-known ability of promoting their electro-oxidation/reduction at moderate potentials, therefore avoiding major interferences.

Despite their great potential to closely mimic natural enzymes through careful engineering, up to now, the use of Pt nanozymes is mostly limited to glucose spectrophotometric detection, with few applications in electrocatalysis. In most cases, Pt nanozymes are coupled with glucose oxidase (GOx) enzyme, in a cascade reaction exploiting their peroxidase-like activity in catalyzing the oxidation of H_2_O_2_ generated by GOx-mediated glucose oxidation. Such systems do not overcome the above-mentioned issues related to protein fragility as they still require the presence of a natural enzyme. Moreover, the need of additional chromogenic substances for glucose optical detection creates further problems to the system. When used in electrocatalytic applications, severe barriers to establish electrochemical communication between the electrode and the nanozyme are often encountered possibly due to electron transfer resistance at the electrode interface introduced by nanozyme coatings or binding matrices used for nanozyme linkage to the electrode. For overcoming these difficulties, Pt nanomaterials are often integrated with other components, as metal-organic-frameworks, redox mediators, and conductive polymer matrices [17], whose assembly often needs complex and long procedures. Finally, in some cases [16], the claimed enzyme mimicking ability is not supported by the evaluation of kinetic parameters necessary to make a comparison with natural enzymes.

When used in electrocatalytic field, the performances of Pt nanozymes are strongly influenced by three main parameters, namely size/shape, coating, and surface properties [18–26]. While sizes in the range of few nanometers are necessary to mimic natural enzyme specificity and improve performance [27–30], shapes regulate the surface structure, i.e., the arrangement of the atoms at the surface [31] which has been demonstrated to strongly determine the surface properties of nanosized Pt in several applications including glucose oxidation [32, 33]. As for the coating layer effect on Pt-based nanocatalysts, it has been reported that the surface coating can deteriorate the enzymatic properties of nanomaterials, posing a focus on nanoparticle synthetic protocols and subsequent modification [18].

A strong control of the surface structure of the material is thus recognized as a key point for closely mimicking natural enzyme [31, 34]. Nonetheless, although the effects of surface structures on the electrocatalytic glucose oxidation are reported since the first investigations on single-crystal Pt electrodes [35, 36], there are few studies on the use of Pt nanozymes with defined surface structures [16]. Single-crystal electrodes have been demonstrated since long to have much higher activity for glucose electrooxidation than polycrystalline Pt, with the Pt (111) facets being less sensitive to poison formation and more active in terms of peak current than Pt (100) and Pt (110) [37]. Despite its importance, such a structural sensitivity has been only scarcely considered so far, as spherical isotropic Pt NPs, enclosed by equally developed crystallographic planes have been mainly used in glucose electro-oxidation.

**Scheme 1:**
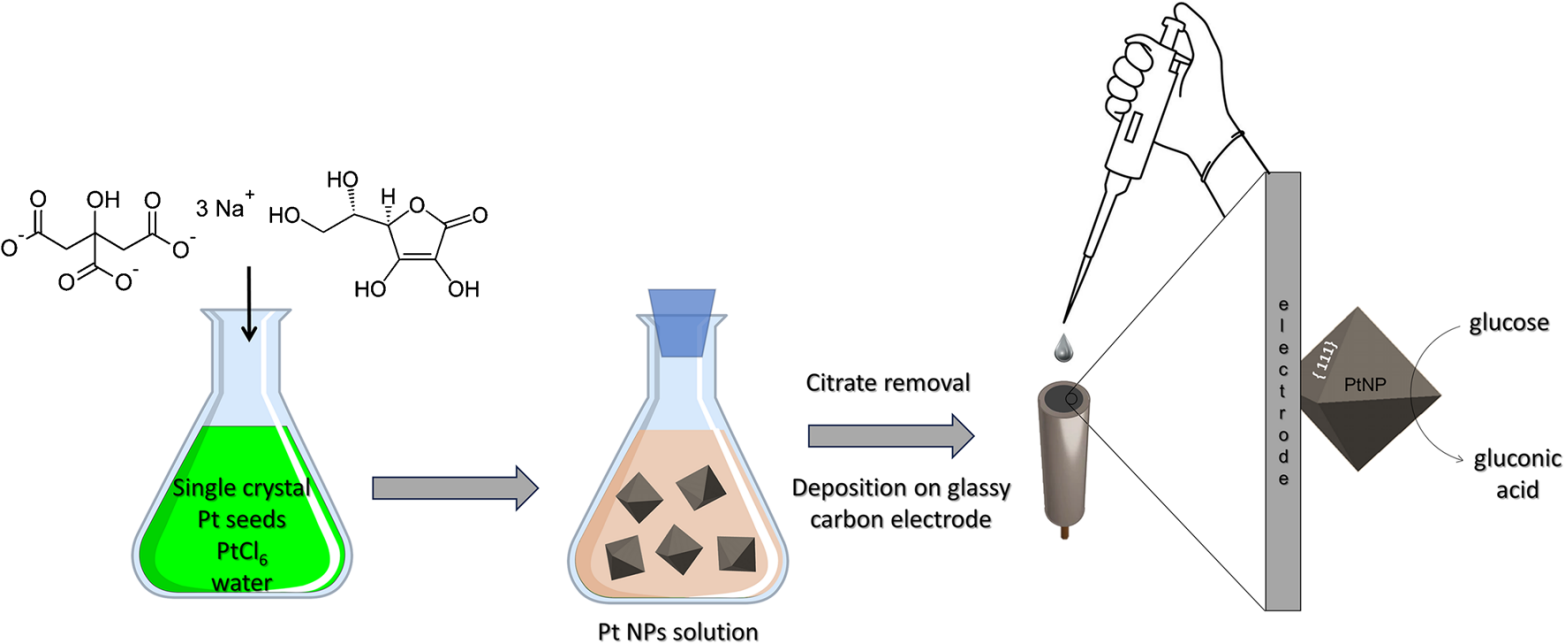
Schematic representation of the synthesis protocol of octahedral Pt NPs and their use for glassy carbon electrode functionalization

In this study, shape-controlled single-crystal octahedral nanoparticles characterized by well-defined extended {111} surface facets and with low polydispersity in size (7 nm and 19 nm) closely mimic GOx enzyme **(**Scheme 1**)**. This is achieved by strictly controlling the surface structure while avoiding the use of polymers and surfactants. Such synthetic design preserves enzymatic-like properties [18] and enhances nanoparticle stability while offering the unique advantage of an easy deposition on the electrode surface.

The mimicking ability of glucose oxidase is proven by the analysis of the steady-state kinetics of catalytic glucose electrooxidation, evidencing a high affinity for glucose, comparable to the natural enzyme. Tests in spiked saliva samples demonstrate the potential of Pt nanozymes in non-invasive hyperglycemia monitoring in saliva, which represents the ideal biological fluid for glucose detection, on the basis of the clear evidence of a direct correlation between blood and salivary glucose [38], allowing to overcome common approaches still relying on enzyme-catalyzed, invasive, painful, and repetitive finger-prick tests.

## Experimental section

### Synthesis of quasi-octahedral 7 nm and 19 nm single-crystal Pt nanoparticles

Pt quasi-octahedral single-crystal seeds were synthesized by following the method reported in our previous report [39]. Further details of the synthetic protocols are reported in the supporting info.

### TEM characterization

For TEM analyses, a small volume of the Pt NP suspensions was drop-cast onto a carbon-coated Cu grid. Bright-field TEM (BF-TEM) images of the Pt NP samples onto carbon-coated Cu grids were acquired using a microscope (JEOL JEM-1011) with a thermionic source (W filament), operating at 100 kV. High-angle annular dark field-scanning TEM (HAADF-STEM) imaging was carried out on a FEI Tecnai G^2^ F20 TEM (Schottky emitter), operated at 200 kV. High-resolution TEM (HR-TEM) images were acquired on an image-Cs-corrected JEOL JEM-2200FS TEM (Schottky emitter), with in-column filter (Ω-type), operated at 200 kV. Further details are reported in the supporting info.

### Deposition of 7 nm and 19 Pt nanoparticles on glassy carbon electrode

Pt nanoparticle deposition on the electrode was carried out as reported elsewhere [40]. Further details are reported in the supporting info.

### SEM and XPS characterization

SEM characterization of glassy carbon substrate surface covered by Pt nanozymes was performed by using JEOL JSM-7500FA microscope.

XPS analysis was performed using an AXIS ULTRA DLD (Kratos Analytical) photoelectron spectrometer equipped with a monochromatic AlKα source (1486.6 eV) operated at 150 W (10 kV, 15 mA), as already described in a previous report [41]. Further details are reported in the supporting info.

### Electrochemical characterization

#### Electrochemical instrumentation

Electrochemical characterization was performed using a PalmSens 4 electrochemical interface. This instrument was managed by PSTrace 5.8 software (PalmSens, Houten, Netherlands). Further details are reported in the supporting info.

#### Characterization by cyclic voltammetry (CV)

Both 7 nm and 19 nm Pt NPs deposited on GC electrode were electrochemically characterized by cyclic voltammetry (CV) in H_2_SO_4_ 0.5 M solutions between −0.2 V and 1.2 V, at scan rate 50 mVs^−1^ for the evaluation of Pt NPs electroactivity. Further details are reported in the supporting info.

#### Glucose detection by multiple pulse amperometry (MPAD)

The electrochemical detection of glucose (0.15–17 mM) was amperometrically performed using multiple pulse amperometry (MPAD), by applying a measurement potential of −0.2 V for 0.4 s and subsequently switching the potential from 0.6 V (applied for 0.2 s) to −0.5 V (applied for 0.2 s). Different measurement potentials were also explored (namely, −0.1 V, −0.2 V, and −0.3 V). Further details are reported in the supporting info.

#### Saliva sample analysis

Glucose detection by MPAD was carried out on real saliva sample, collected and processed using a method reported in the literature [42], slightly modified. More details are reported in the supporting info.

## Results and discussion

### Synthesis and characterization of Pt octahedral nanoparticles with high fraction of {111} surface facets

Pt octahedral nanoparticles (also defined as Pt nanozymes in the text) are synthesized by a seed-templated synthesis technique without the use of organic polymers, organic capping ligands or surfactants, or other inorganic salts (Scheme 1).

**Fig. 1:**
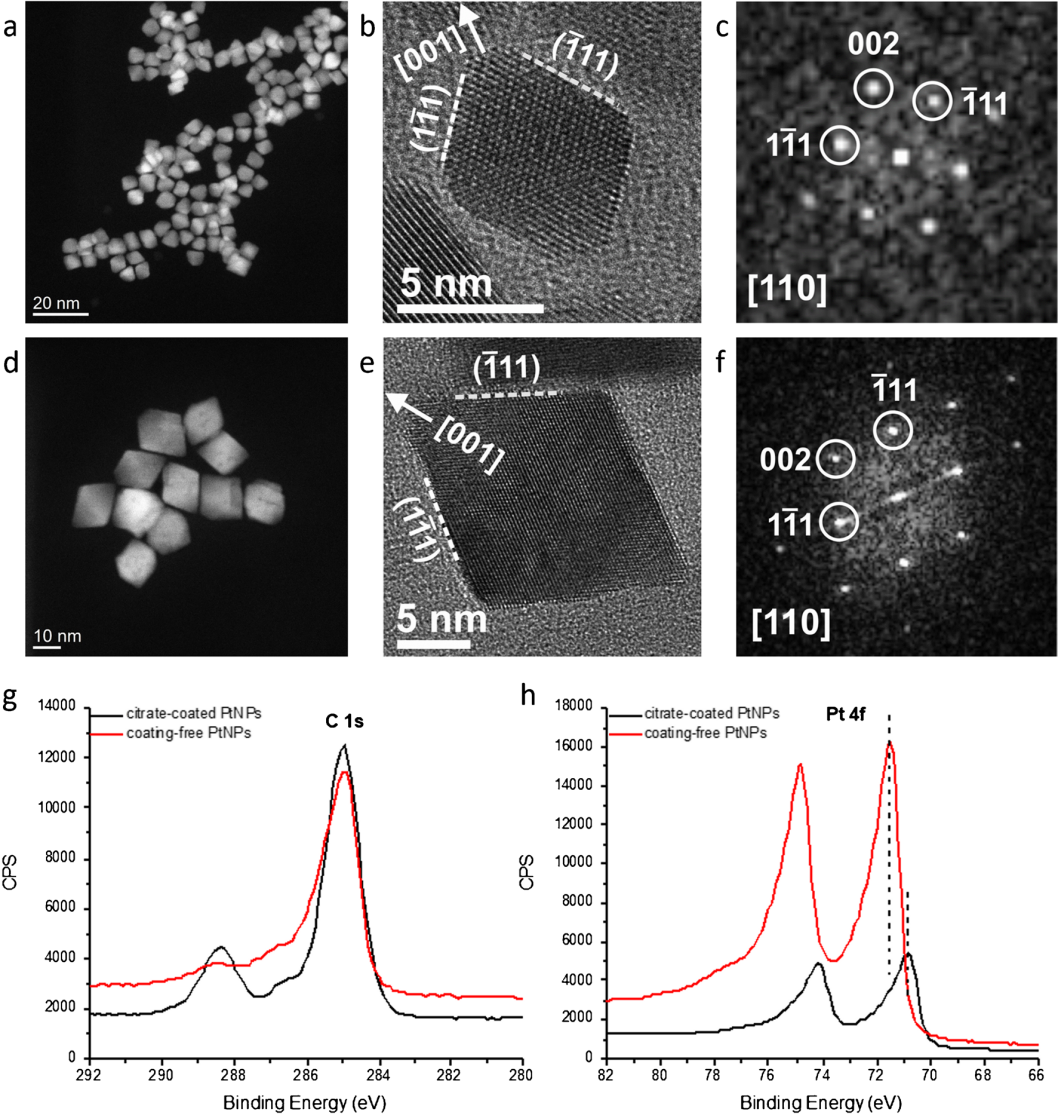
**a**, **d** HAADF-STEM images of the 7 nm (**a**) and 19 nm (**d**) octahedral Pt NPs. **b**, **c**, **e**, **f** HR-TEM images and corresponding fast Fourier transforms (FFTs) of 7 nm (**b**, **c**) and 19 nm (**e**, **f**) octahedral Pt NPs, indexed according to cubic Pt (ICSD 41525). XPS signals of C 1s (**g**) and Pt 4f (**h**) regions recorded on citrate-coated and coating-free 7 nm octahedral Pt NPs

HAADF-STEM and HR-TEM images of the obtained Pt NPs (Fig. 1a–f) clearly show that the shape of Pt NPs is octahedral, mostly enclosed by extended {111} surface facets bordering to neighboring {111} facets and, in part, to {100} facets forming slightly truncated corners. Since it is known that the surface atoms govern catalysis, it can be envisaged that the observed peculiar feature of these Pt NPs, characterized by the predominance of well-defined {111} single crystal, significantly impacts their catalytic behavior. Two different sizes (Fig. 1a–f and Fig S1) have been selected for evaluating size effect on catalytic activity as NP size is another critical property that governs the catalytic processes dictating the geometry in the interaction with molecules. Not only size is crucial but also the polydispersity of the sample is important. For this issue, our synthetic protocol guarantees a narrow size distribution as shown in Figure S1a–f that clearly indicates that Pt NPs have really low polydispersity (around 10% in the case of 7 nm Pt NPs). Finally, SAED patterns of the two samples (7 nm and 19 nm octahedral Pt particles) confirm the formation of phase-pure fcc Pt (Figure S2).

### Deposition on glassy carbon by citrate removal and XPS characterization

As in the case of natural enzyme electrocatalytic applications, when nanozymes are used for catalyzing electrochemical processes, a good communication with the electrode is required for enabling a rapid and efficient electron transfer. For achieving this goal, the assembly of nanozymes with other conducting materials is often proposed [17]. In our case, the direct electrochemical communication with the electrode is established by the clean surface of Pt nanoparticles which is free from contaminants such as polymers and surfactants and is achieved by a simple procedure for the removal of citrate coating. We have designed a protocol that allows to remove the majority of citrate from the NP surface consisting of a simple washing of NPs with NaOH: the increase of the solution pH causes them to lose their stability and deposit on the substrate. In this way, the deposition of the nanoparticles on the electrode consists of easily casting a drop of solution and guarantees a good electrical contact, along with a strong adhesion, responsible for the system stability, as demonstrated below.

The deposition of octahedral Pt NPs on a glassy carbon substrate is revealed by SEM images (Figure S3) proving that a uniform and extensive coverage of the surface has been achieved. The easy citrate coating removal overcomes the problem of the coating remaining on the surface of the nanomaterial after synthesis, and not only ensures easy deposition on the electrode surface, but also the absence of unwanted chemicals that affect the quality of the nanomaterials [43–49]. Although the synthesis of highly dispersed, pure Pt octahedra is an object of an intense research, there are only few reports that avoid the use of polymers, surfactants, and sticky organic molecules in order to promote octahedral Pt growth [39, 50].

Octahedral Pt NPs have been characterized by XPS before and after the treatment with NaOH for the removal of citrate coating. Collected C 1s and Pt 4f spectra are compared in Fig. 1g, h. Looking at the modifications occurring in C 1s signals (Fig. 1g) upon the treatment with the basic solution, it easily emerges a decrease of component at higher binding energy located at about 288.4 eV, attributable to citrate carboxylic moieties [40]. This result shows that the washing procedure with NaOH solution is responsible for the almost complete removal of citrate shell [40]. This is further confirmed by the comparison of Pt 4f signals (Fig. 1h), which show a remarkable increase for the coating-free Pt-NP samples, indicating that the Pt surface is more exposed due to the removal of the citrate coating agent. Also, the observed shift (about 0.7 eV) of Pt 4f signal towards higher binding energy in coating-free samples can be interpreted in the same direction, being related to the removal of citrate coating from the nanoparticle surface. The treatment with NaOH solution provokes a significant decrease of the free carboxylates not bound to platinum atoms (COO^−^−Na^+^) and a consequent increase of COO−Pt moieties [40] , as shown in Figure S4.

Along with the coating-free nature of Pt NPs, their high conductivity can also be inferred from XPS analysis. A metallic behavior was indeed observed in coating-free Pt NPs, which were not affected by any charging effect, contrary to what is commonly observed on insulating or weak conducting samples under an X-ray source, as in the case of citrate-coated samples. Similar XPS results were obtained independently of the size of the nanomaterials under study. This remarkable result reveals that the synthesized Pt nanoparticles with their peculiar surface structure resemble a single {111} crystal possessing a metal-like conductivity.

### Cyclic voltammetry (CV) measurements

Cyclic voltammetry (CV) measurements in 0.5 M H_2_SO_4_ solution were performed on 7 and 19 nm Pt NP samples to evaluate their electrochemical behavior. Figure S5 shows the characteristic voltammetric profiles recorded on both samples which appear very similar with characteristic features associated with the presence of a well-defined (111) preferential surface orientation. In particular, the presence of a small contribution between 0.2 and 0.3 V, a feature related to the anion adsorption on two-dimensionally ordered {111} surface domains, should be remarked. The absence of this signal has been indeed observed on spherical Pt nanoparticles similarly prepared, displaying a voltammetric profile characteristic of a polyoriented Pt surface [40]. This characterization, combined with HR-TEM, further points out the high quality of the present samples (in terms of (111) preferential faceting).

From CV curves in Figure S5, it can be easily appreciated that 7 nm Pt NPs exhibit higher current values in all the investigated potential range, mainly in the so-called “hydrogen region” between −0.25 and 0.1 V where hydrogen adsorption/desorption processes occur, and at higher potential values due to platinum redox process. This is due to the size effect determining higher surface exposure on a smaller-size system.

Data from CV profiles have been used for evaluating the electrochemically active surface area (ECSA) by determining the area of the hydrogen desorption net of the double-layer region. The ECSA was evaluated as already reported in the literature [40]. Further details are reported in the supporting info. ECSA values equal to (5.26 ± 0.32) and (2.85 ± 0.17) cm^2^ mg^−1^ for 7 and 19 nm Pt NPs, respectively, have been estimated. Smaller NP size evidently determines a significant increase of the surface area and, in turn, a higher number of active sites for redox reactions. Interestingly, recorded CV profiles remain almost unmodified even after a long series of cycles (e.g., 40 cycles) proving the high adhesion of NPs to the electrode surface, which, in turn, is due to the coating-free surface of nanomaterial. This is confirmed by the same experiments with citrate-capped octahedral Pt NPs, exhibiting poor adhesion to the electrode as low signals ascribable to Pt redox profile, gradually disappearing upon cycling, were observed most likely due to NP leakage into solution.

### Study of nanozyme catalyzed glucose oxidation process: nanozyme size effect

**Fig. 2:**
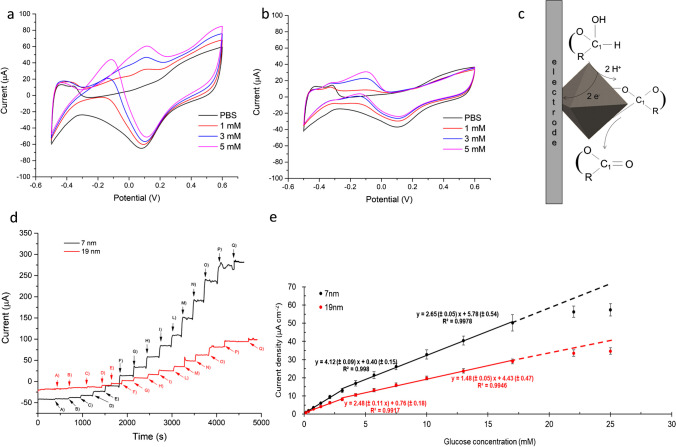
Cyclic voltammograms (1st cycle) recorded on **a** 7 nm and **b** 19 nm octahedral Pt NPs in PBS 0.1 M, pH 7.0 (black curve) and with increasing glucose concentration, scan rate 50 mVs^−1^. **c** Schematic representation of glucose electrooxidation mechanism. **d** Multiple pulse amperometric response on 7 nm (black curve) and 19 nm (red curve) octahedral Pt NPs in PBS 0.1 M, pH 7.0 with glucose 0.05–25 mM (A: 0.05, B: 0.15, C: 0.35, D: 0.75, E: 1.35, F: 2.10, G: 3.10, H: 4.20, I: 5.7, L: 7.5, M: 10, N: 13, O: 17, P: 22, Q: 25 mM); working potential: −0.2V (vs saturated calomel electrode (SCE)). **e** Calibration plots on 7 nm and 19 nm Pt NPs plotting current density (i.e., current normalized respect to ECSA) vs glucose concentrations

Cyclic voltammograms recorded on 7 nm and 19 nm octahedral Pt NPs in the presence of glucose-increasing concentration are shown in Fig. 2a, b. According to what was reported for the oxidation of glucose on (111) single-crystal platinum electrode [36, 37], a single main peak is observed in direct scan on both samples. In the case of 19 nm Pt NPs (Fig. 2b), it is centered at about −0.15 V and is followed by a broad peak with lower currents in the same potential region during the reverse sweep. On 7 nm Pt NPs (Fig. 2a), the peak in the anodic scan appears shifted at a higher potential (about 0.1 V) but is characterized by higher currents. Interestingly, in the reverse scan, the oxidation process proceeds originating a significantly enhanced peak current located at lower potential, showing the high electrocatalytic efficiency of 7 nm Pt NPs.

It should be highlighted that the presence of a single glucose oxidation peak is considered to be a fingerprint of Pt (111) surface, contrarily to what was reported for Pt (100), Pt (110), and on polycrystalline platinum [36, 37] exhibiting two oxidation peaks, located at hydrogen adsorption and double layer regions. The appearance of two distinct glucose oxidation peaks has been indeed observed on isotropic spherical Pt NPs (Figure S6) enclosed by different, equally developed, low-index crystallographic planes characterized by a mixture of different surface facets (Figure S1g, h) [40].

The glucose oxidation mechanism has been long studied [51]. For α- and β-glucose, the hydrogen atom tethered to C_1_ carbon is activated because the acidity of hemiacetalic OH group (pKa = 12.3) is stronger than alcoholic OH (pKa = 16). Thus, the product of electrochemical oxidation of α- and β-glucose is glucono-δ-lactone, which is hydrolyzed into gluconic acid at pH 7.5. The electrochemical oxidation of γ-glucose produces gluconic acid directly. This general mechanism is widely proposed also for Pt surfaces [36, 37] and can be assumed to occur on Pt NPs (Fig. 2c).

Specifically, collected CV data show that on the Pt {111} surface facets of octahedral Pt NPs, the reaction takes place only outside the hydrogen adsorption region, contrarily to spherical Pt NPs (Figures S1g, h and S6) on which it occurs also at the surface almost fully covered by hydrogen. This could partly explain the reported structural sensitivity of glucose oxidation reaction as on octahedral system, it occurs on a “clean” surface possibly determining a higher amount of adsorbed glucose and, therefore, higher electrocatalytic efficiency [36, 37]. It is interesting to observe that on Pt systems exhibiting two glucose oxidation peaks, the second peak is ascribed to the oxidation of adsorbed species formed during glucose oxidation, mainly anions and glucono-δ-lactone [51]. The absence of a second peak on Pt (111) octahedral Pt NPs could thus suggest the absence of strongly bound species and their reduced sensitivity to poison formation. From the comparison of CV data relevant to octahedral Pt NPs—rich in extended {111} single crystal facets—and isotropic spherical Pt NPs—characterized by a mixture of different surface facets—it is thus evident that the peculiar surface structure of octahedral Pt nanoparticles is the key aspect determining their high electrocatalytic activity towards glucose oxidation allowing to achieve higher sensitivity and lower susceptibility to fouling phenomena. These results demonstrate the major effect of surface structure in the electrocatalytic performances of Pt NPs making them the ideal candidate for glucose electrocatalysis.

For further proving the nanozyme size effect, glucose detection on 7 nm and 19 nm Pt octahedral nanozymes has been performed by multiple pulsed amperometric detection (MPAD). MPAD is selected due to its ability of enhancing sensitivity and permitting a continuous electrode surface regeneration while reducing possible detrimental effect due to poison formation [40]. Specifically, the applied MPAD signal consists of three steps: the first at an oxidative potential, i.e., 0.6 V for 0.2 s, the second at −0.5 V for 0.2 s, and the last one at which signal current is measured. The two applied potentials, 0.6 V and −0.5 V, have been selected on the basis of CV experiments for guaranteeing the complete oxidation/reduction of adsorbed species possibly formed during glucose oxidation. Three different measuring potentials have been compared, namely −0.1, −0.2, and −0.3 V, selected on the basis of CV curves in Fig. 2a, leaving other parameters unchanged. CV shows that the electro-oxidation of glucose takes place during both direct and reverse scans, as expected for a catalytic process. Specifically, on the reverse scan, it is evident that there is a remarkable current increase in the potential range between 0 and −0.4 V, with a maximum current increase at −0.2 V. Three values within such potential range have been thus compared as shown in amperometric curves reported in Figure S7**.** By applying −0.2 V, a higher sensitivity with higher currents at each glucose concentration is achieved, together with a higher signal-to-noise ratio, which appropriately directs the choice of such conditions for further investigations.

Results of MPAD on 7 nm and 19 nm octahedral Pt NPs are reported in Fig. 2d, comparing the amperometric responses of both systems to increasing glucose concentrations (0.05–25 mM). What suddenly emerges is the significantly higher sensitivity of 7 nm Pt NPs exhibiting higher current responses at each glucose concentration, thus corroborating the existence of an increased number of Pt active sites. Calibration plots in Fig. 2e show two distinct linear ranges on both systems: the first from 0.36 to 3 mM and the second from 3 to 17 mM. The presence of two linear ranges is a common feature of enzyme and enzyme-like catalytic systems [52–54]. It reveals that peak currents increase linearly with the increase of concentrations at lower concentrations, and then gradually deviate from the linearity tending to be steady, which is similar to enzyme kinetics, indicating oxidase-like characteristic of octahedral Pt NPs.

Interestingly, the sensitivity enhancement achieved by smaller Pt NPs is almost the same on the entire concentration range (namely, 1.7 and 1.8 in the first and in the second linear range, respectively) reflecting the higher electroactive surface area of smaller nanoparticles, being the ratio between ECSA values of two systems equal to 1.8. These results also confirm that the larger electroactive surface area gives the NPs an enhanced ability to interact with glucose, making the system more suitable for electrocatalytic sensing applications. Further investigations were therefore carried out on 7 nm octahedral Pt NPs.

### Study of the nanozyme structural effect on the electrocatalytic detection of glucose

As discussed above, structural effects are known to strongly influence the electrocatalytic performances of Pt-based systems in glucose oxidation. With the aim of proving this key aspect, the amperometric response to glucose of 7 nm octahedral Pt NPs, rich in {111} surface facets, has been compared with that on similarly nanosized Pt systems, namely 4 nm spherical Pt NPs, which are enclosed by different, equally developed, low-index crystallographic planes (Figure S1g, h) [40]. Results shown in Figure S8 clearly demonstrate the higher sensing performances of the octahedral Pt system. Even considering only the first higher-sensitivity linear range, a significantly lower sensitivity is achieved with spherical Pt NPs, with a 53% sensitivity decrease possibly ascribed to structural effects. This result can be due to both the enhanced glucose interaction with {111} surface facets and to the reduced tendency of Pt (111) sites to adsorb fouling species, as discussed above.

### Study of the nanozyme surface coating effect on the electrocatalytic detection of glucose

One major feature of the proposed system is the demonstrated absence of polymers and surfactants which provide clean nanostructured surfaces. For demonstrating the enhanced catalytic properties ascribable to the coating-free property of Pt NPs, their electrocatalytic activity has been compared with that of similarly sized octahedral Pt NPs coated with a PVP shell. From data reported in Figure S9, the superior performances of coating-free Pt NPs clearly emerge. PVP-coated nanoparticles not only exhibit negligible current responses but tend to rapidly reach a saturation condition which determines the absence of current responses for glucose concentration higher than 1 mM, as shown in the calibration plot (Figure S9, inset). Low sensitivity and poor linearity are exhibited by PVP-coated nanoparticles in the whole concentration range. Such limited catalytic properties can be attributed to the presence of organic coating which evidently acts reducing the number of catalytically active sites available for glucose oxidation.

### GOx-mimicking activity of 7 nm octahedral Pt nanozyme: evaluation of analytical performances in glucose electrocatalytic oxidation

The ability of octahedral Pt nanoparticles to act as GOx-mimicking nanozymes has been proved by testing their analytical performances in glucose electrooxidation with the aim to make a quantitative evaluation of parameters that can effectively foster the natural enzyme replacement in glucose monitoring practices.

The limit of detection [55, 56] (LOD, estimated as 3d/m, where d is the residual standard deviation of the linear regression and m is the sensitivity of calibration plot) for 7 nm octahedral Pt nanoparticles is equal to 110 μM (1.98 mg/dL), as confirmed by sensor tests at this glucose concentration. This value opens the possibility to test glucose concentration in the saliva of diabetic patients as the salivary basal physiological values of glucose are below 2 mg/dL while the hyperglycemic spikes are above 4 and 6 mg/dL [38, 57] thus allowing the perspective application of the nanozyme-based sensor in frequent and non-invasive monitoring of hyperglycaemia in saliva.

**Fig. 3:**
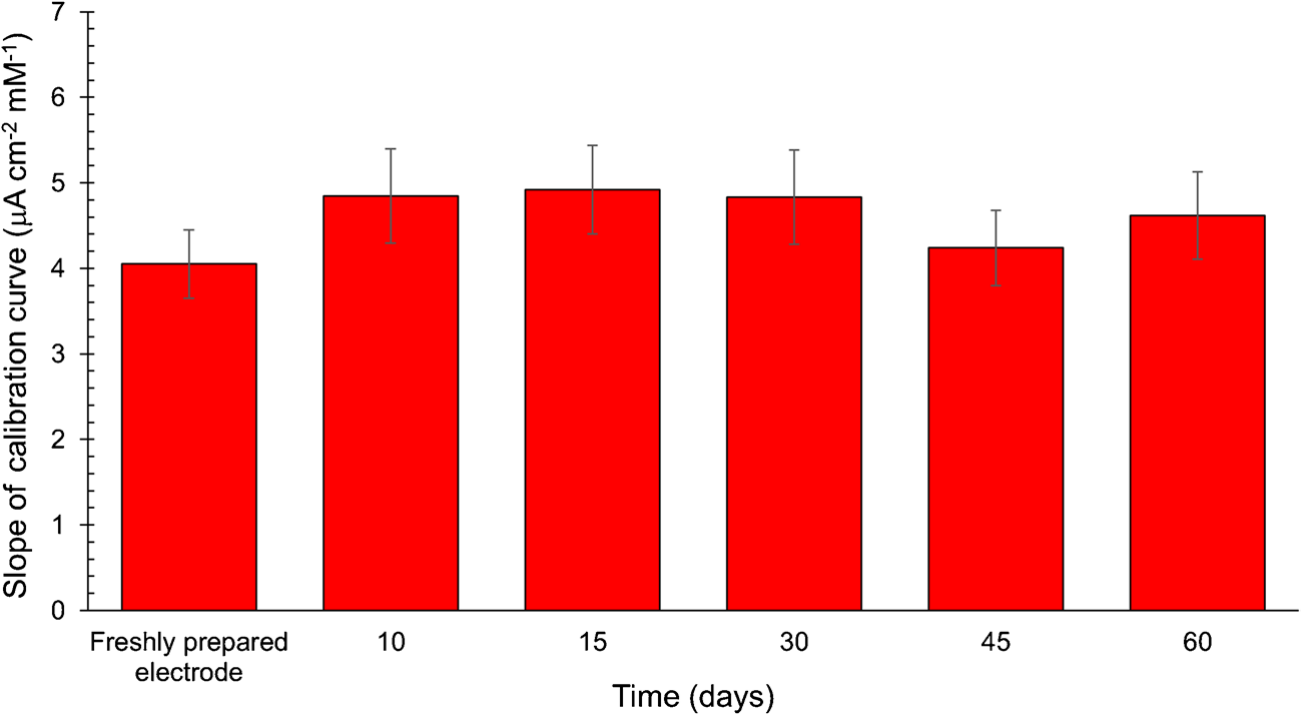
Stability of the octahedral Pt nanozymes sensor response (60 days’ timescale). At each time, MPAD is carried out in the presence of glucose 0.05–25 mM and sensor performances are compared in terms of slope of calibration plot

A good reproducibility has been achieved, with an average relative standard deviation (RSD) value of 9.1% (*n* = 3). Similarly, a low intra-sensor variability has been observed, with a percentage RSD value of 6.6% (*n* = 3). Moreover, the sensor is long-lasting and can be used for at least 60 days, with only 7.8% response variability. This is clearly proven by comparing the freshly prepared sensor response with that observed after 10, 15, 30, 45, and 60 days (Fig. 3) observing only negligible variations and thus a good time stability.

**Fig. 4:**
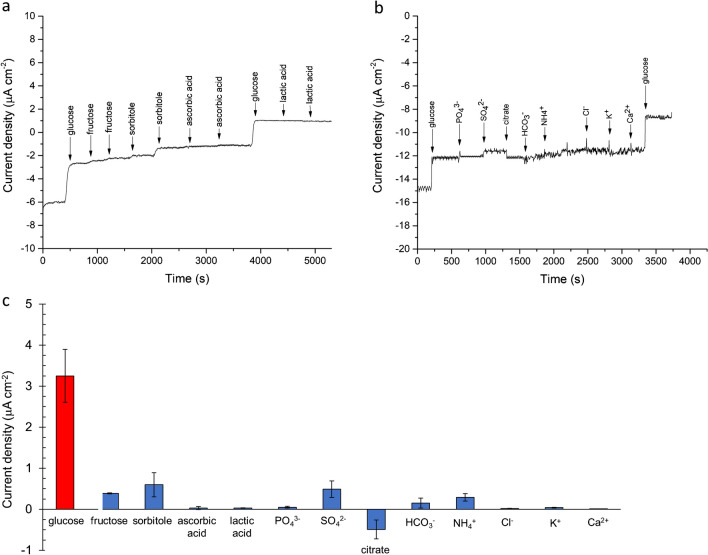
MPAD curve on 7 nm octahedral Pt NPs in PBS 0.1 M, pH 7.0 in the presence of **a** glucose, fructose, sorbitol, ascorbic acid and lactic acid, each at 1 mM, and **b** PO_4_^3−^, SO_4_^2−^, citrate, HCO_3_^−^, NH_4_^+^; Cl^−^, K^+^, and Ca^2+^, each at 1 mM. **c** Graph-bar reporting 7 nm octahedral Pt NPs MPAD response to species tested in selectivity experiments. Amperometric density currents recorded at 1 mM

To establish the selectivity of Pt nanozymes sensor response to glucose, MPAD measurements have been performed in the presence of interfering organic molecules, namely fructose, sorbitol, ascorbic acid, and lactic acid, each at 1 mM. Results are shown in Fig. 4a–c. The system exhibits only minor responses to all tested species, suggesting that the nanozyme has a good selectivity for glucose oxidation, possibly ascribed to the applied potential, at which the oxidation of tested interferent is negligible. Also, the effect of different ionic species on sensor response has been tested (Fig. 4b, c) observing that the presence of both anions and cations possibly coexisting with glucose in real matrices as saliva does not appreciably influence sensor response to glucose.

Glucose detection by the nanozyme-based sensor was performed in spiked saliva samples. As shown in Table 1, the obtained recoveries values are in the range of 92–98%, demonstrating that the system can be employed for glucose detection in real saliva samples.

**Table 1:** Recovery percentage values of glucose in spiked saliva samples

Added (mM)	Found (mM)	Recovery%
0.35	0.34 ± 0.04	98
0.75	0.69 ± 0.05	92

**Fig. 5:**
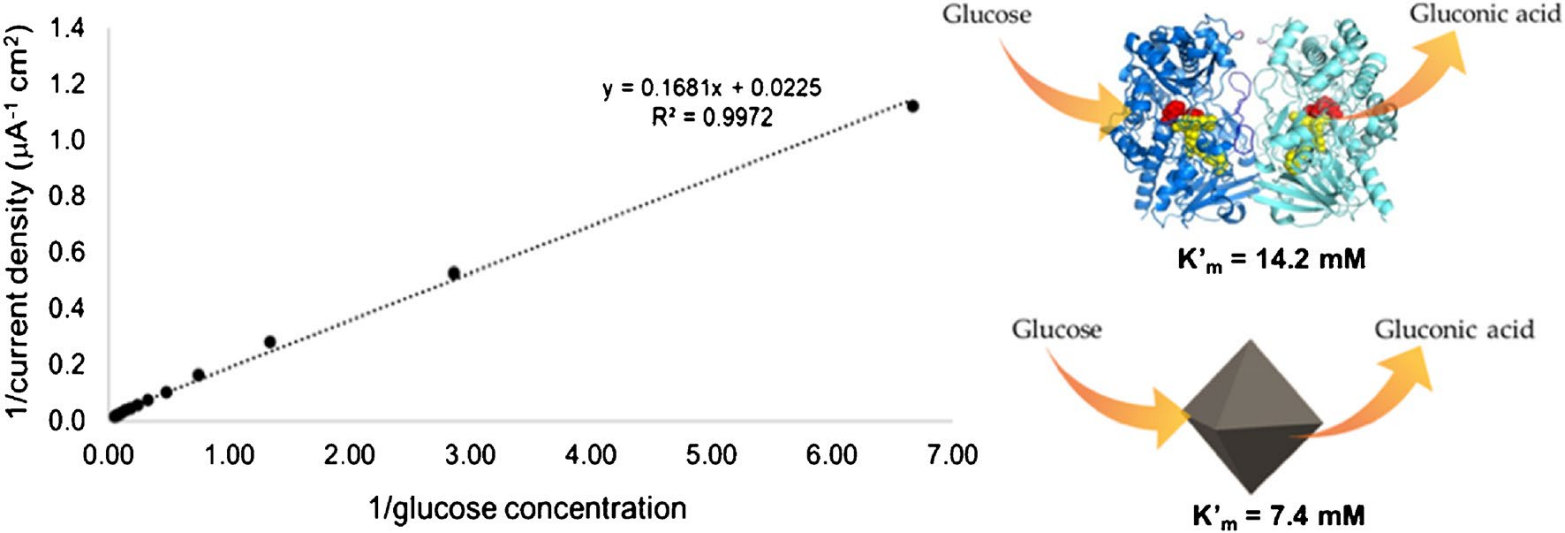
Lineweaver-Burk plot of calibration data presented in Fig. 2e

In order to evaluate the enzyme-like behavior of the proposed nanozyme, kinetic parameters, namely *i*_max_ and *K*_m_, have been estimated by using the Lineweaver-Burk linearization of Michaelis-Menten equation as follows (Eq. 1):

1 $$\frac1i=\frac{C+K{'}_m}{\;i_{max}C}=\frac{K{'}_m}{i_{max}}\frac1C+\frac1{i_{max}}$$

where *i* is the steady-state current, *i*_max_ is the maximum current under saturating substrate conditions, *K*′_m_ is the apparent Michaelis-Menten constant, and *C* is the concentration of glucose in solution [58]. By employing calibration data (Fig. 2e) and Eq. 1, the results shown in Fig. 5 are obtained.

From the data fitting, *K*′_m_ = 7.4 mM and a maximum current density of 44.5 μA/cm^2^, corresponding to *i*_max_ = 1.87 μA, were obtained. The Michaelis–Menten constant (*K*_m_) is a key parameter that indicates the enzyme affinity towards its substrate, with a lower *K*_m_ value typically referring to a stronger affinity. The value of *K*_m_ also represents the substrate concentration at which the enzyme operates at one-half of its *v*_max_. If an enzyme has a low *K*_m_ value, it achieves a maximal catalytic efficiency at a low substrate concentration [59]. In our case, the estimated value of apparent *K*_m_ is significantly comparable to the reported *K*_m_ value reported for glucose-oxidase (14.2 mM) [58]. This result clearly suggests that octahedral Pt nanozyme shows an excellent mimic activity with a high affinity towards glucose comparable to that of the natural enzyme.

Such results, together with glucose sensing performances, reveal the strength of the proposed sensor representing an artificial system mimicking a natural enzyme with the advantage of reduced costs and higher stability. The observed limit of detection, possibly representing a limitation in comparison with the natural enzyme as it restricts the application in physiologic conditions, makes the system indeed suitable for monitoring hyperglycemia in saliva. To the best of our knowledge, this is the first report that proves that well-defined and extended {111} surface facets on octahedral nanoparticles closely mimic and replace GOx enzyme (Table S1).

## Conclusion

Nanozymes represent a great opportunity to overcome the limitations of natural enzymes, providing a reliable, low cost, and highly durable alternative for biomedical and diagnostic applications. Here we describe nanozymes based on shape controlled single-crystal octahedral nanoparticles characterized by well-defined extended {111} surface facets, low polydispersity in size, and an easy-to-remove coating, able to mimic glucose oxidase enzyme, with similar *K*_m_ value. Glucose detection shows a wide linear response, interesting limit of detection and excellent reproducibility coupled with long-term stability. The performance of the device in saliva samples reveals the applicability of Pt nanozymes in non-invasive detection of hyperglycemia thus leveraging the unique peculiarity of saliva of being largely available and easily accessible in a non-invasive and pain-free way.

### Supplementary information

ESM 1 (DOCX 6.00 MB)
