# Supplementary material for: Nanozymes based on octahedral platinum nanocrystals with {111} surface facets: glucose oxidase mimicking activity in electrochemical sensors
Source: Mikrochim Acta. 2023 Sep 30;190(10):425. doi: 10.1007/s00604-023-05992-9 (PMC10543470; doi:10.1007/s00604-023-05992-9)
Supplement: Supplementary file 1 — (DOCX 6.00 MB) [file 604_2023_5992_MOESM1_ESM.docx]

**SUPPORTING INFORMATION**

**Nanozymes based on octahedral platinum nanocrystals with {111} surface facets: glucose oxidase mimicking activity in electrochemical sensors**

**Elisabetta Mazzotta*^a^, Tiziano Di Giulio^a^, Valentina Mastronardi^b^, Rosaria Brescia^c^,**

**Pier Paolo Pompa^b^, Mauro Moglianetti*^b,d,e^, Cosimino Malitesta^a^**

*^a^Laboratorio di Chimica Analitica, Dipartimento di Scienze e Tecnologie Biologiche e Ambientali (Di.S.Te.B.A.), Università del Salento, 73100 Lecce, Italy*

*^b^Istituto Italiano di Tecnologia, Nanobiointeractions&Nanodiagnostics, Via Morego 30, 16163 Genova, Italy*

*^c^Electron Microscopy Facility, Istituto Italiano di Tecnologia, Via Morego 30, 16163 Genova, Italy*

*^d^Istituto Italiano di Tecnologia, Centre for Cultural Heritage Technology (CCHT@Ca' Foscari), Via Torino 155, 30172 Venice, Italy*

*^e^HiQ-Nano srl, Via Barsanti, 1, 73010 Arnesano (LE), Italy*

*Corresponding authors: [elisabetta.mazzotta@unisalento.it](mailto:elisabetta.mazzotta@unisalento.it) , [mauro.moglianetti@iit.it](mailto:mauro.moglianetti@iit.it)

Materials

Sodium hydroxide, [≥98%, pellets (anhydrous)](https://www.sigmaaldrich.com/IT/it/product/sigald/s5881), monosodium phosphate, ≥99.0% (MSP), NaH_2_PO_4_, and disodium phosphate, ≥99.0% (DSP), Na_2_HPO_4_, were provided from Honeywell Fluka (College Park, GA, USA). Sodium chloride, ≥99.5% (NaCl), potassium chloride, ≥99.0% (KCl), calcium chloride ≥99.0%, powder, (anhydrous) (CaCl_2_), sodium bicarbonate, ≥99.5%, powder (NaHCO_3_), sodium citrate, ≥99.5%, tribasic dihydrate, ammonium chloride, ≥99.5% (NH_4_Cl), sodium sulfate, ≥99.0%, powder (anhydrous) (Na_2_SO_4_), sodium phosphate (Na_3_PO_4_), were obtained from Alfa Aesar (Kandel, Germany). D-glucose, ≥99.5% (Dextrose anhydrous), D-fructose, ≥99.0% D-sorbitol, ≥99.0% and lactic acid, 85% (w/w), syrup, were provided by Sigma-Aldrich.Chloroplatinic acid hexahydrate BioXtra, L-ascorbic acid BioXtra, sodium citrate tribasic dihydrate BioUltra, sodium borohydride ≥99.5%, polyvinylpyrrolidone (PVP) ≥99.5% and citric acid anhydrous ≥99.5% were bought from Merck/Sigma-Aldrich and used as received. Phosphate buffer saline (PBS) solutions (0.1 M, pH 7.0), were prepared by dissolution of the commercial MSP and DSP in appropriate proportions, adding NaOH 0.5 M to adjust the final pH. Glucose stock solution (1 M) was prepared using phosphate buffer (PBS) 0.1 M at pH 7. Stock solutions of fructose, sorbitol, ascorbic acid, lactic acid, sodium chloride (NaCl), potassium chloride (KCl), calcium chloride (CaCl_2_), sodium bicarbonate (NaHCO_3_), sodium citrate, ammonium chloride (NH_4_Cl), sodium sulfate (Na_2_SO_4_), sodium phosphate (Na_3_PO_4_) (1 M), were prepared in PBS 0.1 M at pH 7 and used as interfering molecules for glucose.

Synthesis of quasi-octahedral 7 nm and 19 nm single crystal Pt nanoparticles

Pt quasi-octahedral single crystal seeds were synthesized by following the protocol reported in our previous report.^1^

*19 nm octahedral NPs Pt synthetic procedure*

The synthesis was performed in a sealed glass container (ACE glass pressure reactor with Teflon cap). 3.5 ml of platinum nanoparticles seed (synthesized following a previously developed protocol) were added to 84 ml of MilliQ water. 0.11 mL of hexachloroplatinic acid aqueous solution (0.5 M) was added together with 1.5 ml of 1% sodium citrate and 1.25% L-ascorbic acid solution. The vessel was then sealed, immersed in an oil bath and brought to 98 °C in 20 minutes. The reaction was kept at these conditions for 1 hour under magnetic stirring at moderate rate (500 rpm on Velp Scientifca Arec X Heating magnetic stirrer). The vessel was then removed from the oil bath and left to cool under stirring for another hour.

*7 nm octahedral NPs Pt synthetic procedure*

7 octahedral nanoparticles were synthesized following the protocol presented in the previous sections. 2 nm size seeds and lower amount of hexachloroplatinic acid hexahydrate were used to reduce the growth while keeping the control on the synthesis.

3.5 ml of platinum nanoparticles seeds solution with average size around 2 nm was added to 84 ml of MilliQ water. 55 μL of hexachloroplatinic acid aqueous solution (0.5 M) was added together with 1.5 ml of 1% sodium citrate and 1.25% L-ascorbic acid solution. The vessel was then sealed, immersed in an oil bath and brought to 98 °C in 20 minutes. The reaction was kept at these conditions for 1 hour under magnetic stirring at moderate rate (500 rpm on Velp Scientifca Arec X Heating magnetic stirrer). The vessel was then removed from the oil bath and left to cool under stirring for another hour.

**Scale-up of nanoparticle synthesis using Flexiwave Microwave Reactor (Milestone srl)**

*19 nm octahedral NPs Pt scale-up method*

The synthesis was performed in a microwave reactor, using the multi-vessel setup (Flexiwave Microwave Reactor (Milestone srl)). 3 ml of platinum nanoparticles seed with average size around 3 nm (synthesized following a previously developed protocol) were added to 87 ml of MilliQ water. 108 microliter of hexachloroplatinic acid aqueous solution (0.5 M) was added together with 1.5 ml of 1% sodium citrate and 1.25% L-ascorbic acid solution. The 15 vessels were then sealed, placed within the microwave chamber and brought to 105 °C in 20 minutes. The reaction was kept at these conditions for 1 hour under magnetic stirring at moderate rate (70% as indicated on the control panel).

7 shaped nanoparticles were synthesized following the protocol presented in the previous sections. 2 nm size seeds and lower amount of hexachloroplatinic acid solution were used to reduce the growth while keeping the control on the synthesis.

*7 nm octahedral Nps Pt scale-up method*

3 ml of platinum nanoparticles seed with average size around 2 nm were added to 87 ml of MilliQ water. 36 microliter of hexachloroplatinic acid aqueous solution (0.5 M) was added together with 1.5 ml of 1% sodium citrate and 1.25% L-ascorbic acid solution. The 15 vessels were then sealed, placed within the microwave chamber and brought to 105 °C in 20 minutes. The reaction was kept at these conditions for 1 hour under magnetic stirring at moderate rate (70% as indicated on the control panel).

**Purification procedure**

After being cooled to room temperature, the Pt NPs were extensively washed using 3K Amicon filters (Merck) with 2 mM sodium citrate solution.

The procedure is as follows:

- The reaction solution (by using 2 Amicon filters, 50 ml per Amicon filter) is reduced to 3 mL by using Amicon filters with around four centrifuge cycles (4000 g for half an hour).
- The remaining 3 ml solution (in each Amicon filter) is then washed at least three times using the same Amicon filters by adding each time 8 ml sodium citrate solution (2mM) and then centrifuging (4000 g for half an hour).

PVP-coated nanoparticles were obtained by adding 40K PVP at the end of the reaction at a concentration of 10 mg/L and left stirring for a least 1 hour at room temperature. Extensive washing with 10K Amicon filters to remove the unbound polymer was performed.

**Transmission electron microscopy (TEM) characterization**

For TEM analyses, a small volume of the Pt NPs suspensions was drop-cast onto a carbon-coated Cu grid. Bright-field TEM (BF-TEM) images of the Pt NPs samples were acquired using a JEOL JEM-1011 microscope with a thermionic source (W filament) and operated at 100 kV. Selected-area electron diffraction (SAED) and high-angle annular dark field-scanning TEM (HAADF-STEM) imaging was carried out on a FEI Tecnai G^2^ F20 TEM, operated at 200 kV. The lateral size of the nanocrystals was obtained by user-imposed thresholding on the HAADF-STEM images followed by automatic measurement of the Feret's diameter using the ImageJ software.^2^ High-resolution TEM (HR-TEM) images were acquired on an image-Cs-corrected JEOL JEM-2200FS TEM (Schottky emitter), with in-column filter (Ω-type), operated at 200 kV. In order to slow down carbon contamination upon electron beam irradiation, regions of interest were exposed to a relatively low dose rate (~70 electrons/(Å^2^ s), 1 order of magnitude lower than typically used for the CCD camera) and HR-TEM images were acquired using a direct electron detection camera (K2 Summit, Gatan), in super-resolution mode. Each HRTEM image shown here is obtained from a (170 nm)^2^ frame. Each of these frames is obtained by summing a stack of aligned frames, each obtained by short exposure (0.3 s), with a total acquisition time of 12 s.

**Deposition of 7 nm and 19 nm single crystal Pt nanoparticles on glassy carbon electrode**

Pt nanoparticles deposition on the electrode was carried out as reported elsewhere.^3^ Briefly, NaOH (about 0.2 g) was added to 1 mL of Pt NPs suspension and diluted with 4 mL of water. Once the precipitation of the nanoparticles is completed, the supernatant was removed, and 5 mL of water added. The procedure was repeated for at least three times. The NPs sample was then sonicated for at least 10 minutes and 45 μL was deposited on the electrode surface, which was then left in air until complete solvent evaporation.

**XPS characterization**

XPS measurements were recorded under the experimental conditions reported in a previous report with an AXIS ULTRA DLD (Kratos Analytical) photoelectron spectrometer using a monochromatic AlKα source (1486.6 eV) operated at 150 W (10 kV, 15 mA).^4^ Survey scan spectra were recorded using a pass energy of 160 eV and a 1 eV step. High resolution spectra were acquired using a pass energy of 20 eV and a 0.1 eV step. The area of analysis has the following dimensions: ~700 μm*300 μm. During the data acquisition a system of neutralization of the charge has been employed. Data analysis was done by using CasaXPS Release 2.3.16 software. The binding energy (BE) scale was referenced to the Au 4f7/2 peak at 84.0 eV. High resolution spectra were fitted using Shirley background and GL(30) lineshape (a combination of Gaussian 70% and Lorentzian 30%). For quantitative analysis, the relative sensitivity factors present in the library of CasaXPS for the areas of the signals were used. Surface charging was corrected considering adventitious C 1s (binding energies (BE=285 eV).^4^ Pt NPs samples after washing for citrate removal were analyzed as a conducting specimen. For XPS analysis, samples were deposited by casting on a glassy carbon sheet.

**Electrochemical experiments**

***Electrochemical apparatus***

Electrochemical characterization was performed with a portable potentiostat/galvanostat, PalmSens. This device was controlled by the PSTrace 5.8 software (PalmSens, Houten, Netherlands). A single compartment glass cell was used, containing three electrodes: a saturated calomel electrode (SCE) as reference electrode, an auxiliary electrode consisting of a platinum wire and a commercial glassy carbon electrode of 3 mm in diameter as working electrode. All electrodes were purchased from CH Instruments (Tennison Hill Drive, AU, USA). All electrochemical measurements were performed without the deaeration of the solutions.

***Cyclic Voltammetry characterization***

Both 7 nm and 19 nm Pt NPs deposited on GC electrode were characterized electrochemically by cyclic voltammetry (CV) in H_2_SO_4_ 0.5 M between -0.2 V and 1.2 V, at scan rate 50 mVs^-1^ for the evaluation of Pt NPs electroactivity. Both systems were also characterized by CV in PBS 0.1 M pH 7.0 between -0.5 V and 0.6 V, at scan rate 50 mVs^-1^, in the presence of increasing glucose concentrations.

***Glucose detection by Multiple Pulse Amperometry (MPAD)***

The analytical detection of glucose (0.15 – 17 mM) was performed amperometrically, by multiple pulse amperometry (MPAD) by applying a potential of -0.2 V (measurement potential, hold for 0.4 s) and subsequently switching the potential from 0.6 V (applied for 0.2 s) to -0.5 V (applied for 0.2 s) versus a SCE electrode. Different measurement potentials were also tested (namely, -0.1 V, -0.2 V and -0.3 V). For comparison, amperometric detection at a fixed potential of -0.2 V was also performed. MPAD measurements were performed on three freshly prepared electrodes for calibration curve and for the evaluation of sensors reproducibility, which has been estimated by comparing the slope of calibration curve obtained on three electrodes.

Considering the performances achieved with 7 nm Pt NPs system, further measurements were carried out using this nanomaterial. Selectivity studies were carried out by analyzing MPAD response of 7 nm Pt NPs to fructose, sorbitol, ascorbic acid and lactic acid, each at 1 mM. To investigate sensor response repeatability, MPAD experiments were performed in triplicate using the same sensor, extensively washing the electrode in water before a new experiment. 7 nm Pt NPs sensor storage stability was evaluated by analyzing the amperometric response to glucose (0.15 – 17 mM) after 10, 15, 30, 45 and 60 days following sensor preparation, upon storage in PBS 0.1 M, pH 7.0.

***Saliva sample analysis***

Glucose detection by MPAD was performed on real saliva sample, collected and processed using a method reported in literature,^5^ slightly modified. In brief, a saliva sample was collected by a healthy volunteer from our laboratory. The volunteer was instructed to avoid eating or drinking and not to brush teeth for at least 1 h before the saliva collection. Volunteer rinsed the mouth with distilled water to remove cellular debris, then unstimulated whole saliva was collected by spitting. The sample was centrifuged at 3000 rpm for 10 min and supernatant was collected. Later, the supernatant was diluted 10 times with PBS pH 7 and then stored at -20°C. Upon verifying the absence of an appreciable amperometric response on the as-obtained saliva samples, different glucose concentrations (0.25, 0.75 mM) were added and percentage recovery for each tested concentration was evaluated for the matrix effect estimation.

***Evaluation of the Electrochemically Active Surface Area (ECSA)***

The ECSA was evaluated according to equation (1).

$\mathrm{ECSA}\left( \frac{\mathrm{cm}^{2}}{\mathrm{mg}} \right)=\frac{Q_{H}(C)}{210\times{10}^{-6}\left( C \mathrm{cm}^{-2} \right)L_{\mathrm{Pt}}\left( \mathrm{mg} \right)}$ (1)

where Q_H_ (C) is the charge exchanged during the hydrogen desorption on the Pt surface, 210×10^−6^ (Ccm^−2^) represents the charge required to oxidize a monolayer of hydrogen on a smooth Pt surface.[3] L_Pt_ (mg) represents the Pt loading on the electrode and is equal to 0.008 mg, as determined considering the Pt NP concentration in the stock solution, water volume used for suspending NPs after washing steps, and the volume of NP solution deposited on the electrode surface (45 μL).


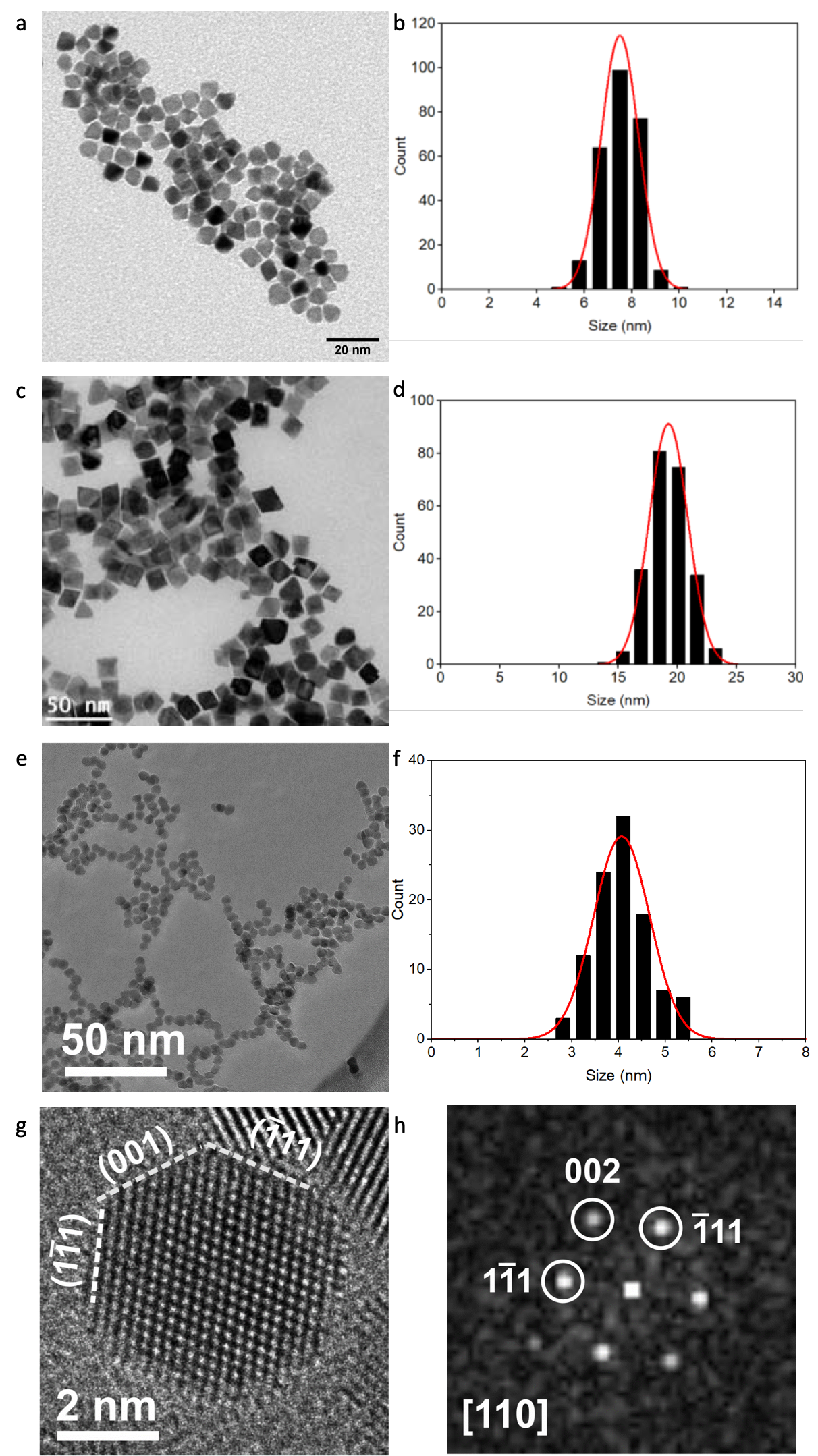


**Figure S1.** BF-TEM images of (a) octahedral 7 nm PtNPs, (c) octahedral 19 nm Pt NPs and (e) spherical 4 nm Pt NPs and relative size distribution equal to (b) 7.4± 0.7 nm, (d) 19.2± 1.6 nm Pt NPs and (f) 4.0 ± 0.6 nm. (g) HR-TEM image and (h) corresponding fast Fourier transform (FFT) of a 4 nm spherical Pt NPs, indexed according to cubic Pt (ICSD 41525).


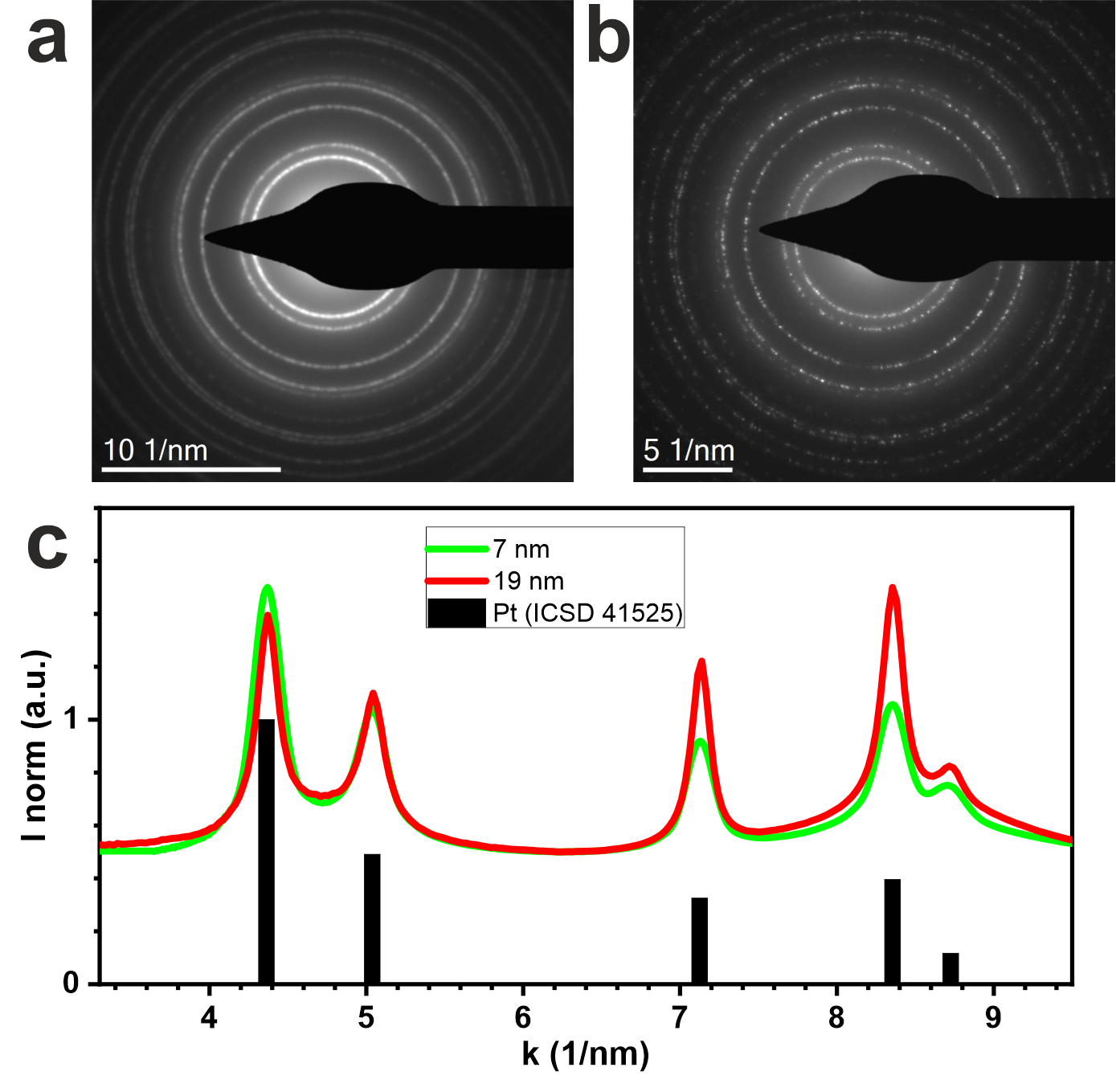


**Figure S2.** SAED patterns collected on (a) 7 nm and (b) 19 nm octahedral Pt NPs and (c) their background-subtracted azimuthal integration, compared with a powder XRD pattern calculated for fcc Pt (ICSD 41525).

**Figure S3.** SEM images of 7 nm PtNPs deposited on a glassy carbon substrate after removal of citrate molecules at different magnifications.

**Figure S4.** Fitted C 1s signals of citrate-coated (left) and coating-free (right) octahedral Pt nanoparticles. Spectra are charging-corrected.

**

**

**Figure S5.** Cyclic voltammograms recorded in H_2_SO_4_ 0.5 M, scan rate 50 mVs^-1^ on 7 nm and 19 nm octahedral Pt NPs.





**Figure S6.** Cyclic voltammograms recorded on 4 nm spherical Pt NPs in PBS 0.1 M, pH 7.0 (black curve) and with increasing glucose concentration, scan rate 50 mVs^-1^.





**Figure S7.** Multiple Pulse Amperometric on 7 nm octahedral PtNPs in PBS 0.1 M, pH 7.0 with glucose 1-12.5 mM under different measuring potentials.





**Figure S8.** Multiple Pulse Amperometric on 7 nm octahedral PtNPs (black curve) and 4 nm spherical/polycrystalline PtNPs (cyan curve) in PBS 0.1 M, pH 7.0 with glucose 0.05-25 mM.

**Figure S9.** Multiple Pulse Amperometric on 7 nm octahedral coating-free (black curve) and PVP-coated (red curve) PtNPs in PBS 0.1 M, pH 7.0 with glucose 0.05-25 mM.

**Table S1 -** Reports on Pt nanoparticles as nanozymes for the detection of glucose (colorimetric and electrochemical methods)

| **Electrochemical methods** | | | | | | | | |
| --- | --- | --- | --- | --- | --- | --- | --- | --- |
| Materials | Pt as GOx | Michaelis Menten constant  (μM) | Pt as HRP | Detection limit  (µM) | Sensitivity  (µA mM^−1^ cm^−2^) | Liner range  (mM) | Matrix | Ref. |
| GOx/Pt NPs/graphene | ✘ | ✘ |  | 0.76 | 37 | 0.006–0.7 | Interstitial fluid (ISF) | ^6^ |
| PtNi alloy-graphene/GC electrode | ✘ | ✘ |  | 16 | 24.03 | 0.5–15 | Blood serum | ^7^ |
| PtNi—ERGO modified electrode | ✘ | ✘ |  | 10 | 20.42 | 0.01–35 |  | ^8^ |
| Pt/Ni Nanowire array electrode | ✘ | ✘ |  | 1.5 | 920 | 2×10^−3^–2 |  | ^8^ |
| Hierarchical Pt micro-/nanostructures | ✘ | ✘ |  | 85 | 473 | Up to 3 | Neutral pH media | ^9^ |
| graphene (G)/platinum oxide (PtO)/n-silicon (Si) heterostructur | ✘ | ✘ |  | Non reported | 0.2 | 2–20 |  | ^10^ |
| GOx/Pt NPs/acetic acid treated LIG | ✘ | ✘ |  | 0.3 | 4.622 | Up to 2.1 | Sweat | ^11^ |
| **Colorimetric methods** | | | | | | | | |
| Materials | Pt as GOx | Michaelis Menten constant  (μM) | Pt as HRP | Detection limit  (µM) | Liner range  (mM) | | Matrix | Ref. |
| Pt–DNA complexes | ✘ | ✘ | **✓** | 0.1 | 0.0001-1 | | beverage | ^12^ |
| poly(styrene sulfonate)/Pt-modified graphene nanosheet | ✘ | ✘ | **✓** | 0.0928 | 0.0002-1 | | Serum | ^13^ |
| Au nanorods @ Pt nanodots core/shell nanostructures | ✘ | 26-2.1 | **✓** | 45 | 0.045-1 | | PBS buffer | ^14^ |
| Polyethyleneimine-Stabilized Platinum Nanoparticles | ✘ | ✘ | **✓** | 0.15 | 0.01-5 | | Saliva | ^15^ |
| Pt55-G5MEK50C | ✘ | 1970 |  | 0.1 | 0.001-2 | | Plasma | ^16^ |

# **References**

1. Hornberger, E.; Mastronardi, V.; Brescia, R.; Pompa, P. P.; Klingenhof, M.; Dionigi, F.; Moglianetti, M.; Strasser, P., Seed-Mediated Synthesis and Catalytic ORR Reactivity of Facet-Stable, Monodisperse Platinum Nano-Octahedra. *ACS Applied Energy Materials* **2021,** *4* (9), 9542-9552.

2. Schneider, C. A.; Rasband, W. S.; Eliceiri, K. W., NIH Image to ImageJ: 25 years of image analysis. *Nature Methods* **2012,** *9* (7), 671-675.

3. Mazzotta, E.; Di Giulio, T.; Mastronardi, V.; Pompa, P. P.; Moglianetti, M.; Malitesta, C., Bare Platinum Nanoparticles Deposited on Glassy Carbon Electrodes for Electrocatalytic Detection of Hydrogen Peroxide. *ACS Applied Nano Materials* **2021,** *4* (8), 7650-7662.

4. Rella, S.; Mazzotta, E.; Caroli, A.; De Luca, M.; Bucci, C.; Malitesta, C., Investigation of polydopamine coatings by X-ray Photoelectron Spectroscopy as an effective tool for improving biomolecule conjugation. *Applied Surface Science* **2018,** *447*, 31-39.

5. Caetano, L. P.; Lima, A. P.; Tormin, T. F.; Richter, E. M.; Espindola, F. S.; Botelho, F. V.; Munoz, R. A. A., Carbon-nanotube Modified Screen-printed Electrode for the Simultaneous Determination of Nitrite and Uric Acid in Biological Fluids Using Batch-injection Amperometric Detection. *Electroanalysis* **2018,** *30* (8), 1870-1879.

6. Lipani, L.; Dupont, B. G. R.; Doungmene, F.; Marken, F.; Tyrrell, R. M.; Guy, R. H.; Ilie, A., Non-invasive, transdermal, path-selective and specific glucose monitoring via a graphene-based platform. *Nature Nanotechnology* **2018,** *13* (6), 504-511.

7. Li, R.; Deng, X.; Xia, L., Non-enzymatic sensor for determination of glucose based on PtNi nanoparticles decorated graphene. *Scientific Reports* **2020,** *10* (1), 16788.

8. Wang, G.; He, X.; Wang, L.; Gu, A.; Huang, Y.; Fang, B.; Geng, B.; Zhang, X., Non-enzymatic electrochemical sensing of glucose. *Microchimica Acta* **2013,** *180* (3), 161-186.

9. Unmüssig, T.; Weltin, A.; Urban, S.; Daubinger, P.; Urban, G. A.; Kieninger, J., Non-enzymatic glucose sensing based on hierarchical platinum micro-/nanostructures. *Journal of Electroanalytical Chemistry* **2018,** *816*, 215-222.

10. Sakr, M. A.; Elgammal, K.; Delin, A.; Serry, M., Performance-Enhanced Non-Enzymatic Glucose Sensor Based on Graphene-Heterostructure. *Sensors* **2020,** *20* (1), 145.

11. Yoon, H.; Nah, J.; Kim, H.; Ko, S.; Sharifuzzaman, M.; Barman, S. C.; Xuan, X.; Kim, J.; Park, J. Y., A chemically modified laser-induced porous graphene based flexible and ultrasensitive electrochemical biosensor for sweat glucose detection. *Sensors and Actuators B: Chemical* **2020,** *311*, 127866.

12. Chen, X.; Zhou, X.; Hu, J., Pt–DNA complexes as peroxidase mimetics and their applications in colorimetric detection of H2O2 and glucose. *Analytical Methods* **2012,** *4* (7), 2183-2187.

13. Chen, J.; Ge, J.; Zhang, L.; Li, Z.; Qu, L., Poly(styrene sulfonate) and Pt bifunctionalized graphene nanosheets as an artificial enzyme to construct a colorimetric chemosensor for highly sensitive glucose detection. *Sensors and Actuators B: Chemical* **2016,** *233*, 438-444.

14. Liu, J.; Hu, X.; Hou, S.; Wen, T.; Liu, W.; Zhu, X.; Yin, J.-J.; Wu, X., Au@Pt core/shell nanorods with peroxidase- and ascorbate oxidase-like activities for improved detection of glucose. *Sensors and Actuators B: Chemical* **2012,** *166-167*, 708-714.

15. Cui, Y.; Lai, X.; Liang, B.; Liang, Y.; Sun, H.; Wang, L., Polyethyleneimine-Stabilized Platinum Nanoparticles as Peroxidase Mimic for Colorimetric Detection of Glucose. *ACS Omega* **2020,** *5* (12), 6800-6808.

16. Cui, Y.; Liu, K.; Cui, T.; Liang, B.; Sun, H.; Wang, L., Development of an Ultrasmall and Biocompatible Platinum Nanozyme Encapsulated by Zwitterionic Dendrimer for Highly Sensitive Detection of Glucose. *Langmuir* **2022,** *38* (18), 5568-5578.
